# Alarming Cargo: The Role of Exosomes in Trauma-Induced Inflammation

**DOI:** 10.3390/biom11040522

**Published:** 2021-03-31

**Authors:** Sarah A. Walsh, Benjamin W. Hoyt, Cassie J. Rowe, Devaveena Dey, Thomas A. Davis

**Affiliations:** 1 USU Walter Reed Surgery, Uniformed Services University, Bethesda, MD 20814, USA; sarah.walsh@usuhs.edu (S.A.W.); benjaminwhoyt@gmail.com (B.W.H.); cassie.rowe.ctr@usuhs.edu (C.J.R.); devaveena.dey@gmail.com (D.D.); 2 Henry M. Jackson Foundation for the Advancement of Military Medicine, Inc., Bethesda, MD 20817, USA

**Keywords:** exosomes, extracellular vesicles, intercellular communication, inflammation, trauma

## Abstract

Severe polytraumatic injury initiates a robust immune response. Broad immune dysfunction in patients with such injuries has been well-documented; however, early biomarkers of immune dysfunction post-injury, which are critical for comprehensive intervention and can predict the clinical course of patients, have not been reported. Current circulating markers such as IL-6 and IL-10 are broad, non-specific, and lag behind the clinical course of patients. General blockade of the inflammatory response is detrimental to patients, as a certain degree of regulated inflammation is critical and necessary following trauma. Exosomes, small membrane-bound extracellular vesicles, found in a variety of biofluids, carry within them a complex functional cargo, comprised of coding and non-coding RNAs, proteins, and metabolites. Composition of circulating exosomal cargo is modulated by changes in the intra- and extracellular microenvironment, thereby serving as a homeostasis sensor. With its extensively documented involvement in immune regulation in multiple pathologies, study of exosomal cargo in polytrauma patients can provide critical insights on trauma-specific, temporal immune dysregulation, with tremendous potential to serve as unique biomarkers and therapeutic targets for timely and precise intervention.

## 1. Introduction

Trauma is a leading cause of morbidity and mortality worldwide [1,2,3,4,5]. Polytrauma occurs daily in the civilian population as a result of motor vehicle collisions, falls from height, firearm discharges, mass transit collisions and derailments, industrial workplace injuries, terrorist attacks, and natural disasters [6,7,8,9,10,11,12,13]. Polytraumatic injuries are complex and involve a combination of concomitant insults to multiple body regions and organs including thermal injuries, large open wounds and major tissue loss, traumatic amputation and eye and nervous system injuries, often resulting in hemorrhage, shock, prolonged systemic inflammation, major organ dysfunction/failure, psychiatric issues, and death.

Blast-related polytraumatic injuries caused by explosive munitions are responsible for 72% of North Atlantic Treaty Organization (NATO) coalition combat casualties in the Global War on Terror [14]. Often during their hospital course, these patients develop severe life-threatening inflammation-mediated complications, to include systemic inflammatory response syndrome (SIRS), compensatory anti-inflammatory response syndrome (CARS), acute respiratory distress syndrome (ARDS), pneumonia, sepsis, multi-organ dysfunction syndrome (MODS), and/or multiple organ failure (MOF). These complications are associated with prolonged stays in the intensive care unit (ICU), increased in-hospital mortality, and long-term physical, cognitive, and psychosocial comorbidities [5,15,16,17,18,19,20,21,22,23,24,25,26].

The body’s innate immune response after trauma is triggered by the detection of damage-associated molecular patterns (DAMPs) released by cell stress, injured and/or dying cells or through detection of pathogen-associated molecular patterns (PAMPs) in the event of injury-associated infection [27,28,29,30,31]. Activation of endothelium results from both mechanical stress and the detection of DAMPs and PAMPs, leading to platelet activation and leaky barriers allowing for leukocyte extravasation [32]. DAMPs, PAMPs and inflammatory cytokines activate neutrophils and macrophages which play a central role in pathogen immune surveillance, tissue inflammation, clearance of debris and necrotic tissue, and in the regulation of successive homeostatic processes involved in tissue repair, healing, and tissue regeneration [26]. Under normal conditions, the immune response is predictable and transient, and well-regulated by a controlled balance of pro- and anti-inflammatory mediators. Trauma-induced inflammation can be a double-edged sword. A “cytokine storm” resulting from the exaggerated production and dysregulation of pro-inflammatory cytokines can result in deleterious systemic inflammation causing further tissue damage, end organ injury, and increased mortality [4,5,24,33]. Therefore, identification of reliable biomarkers of aberrant inflammation early post-injury to predict clinical outcomes and guide treatment is critical. This requires a thorough understanding of the complex local and systemic communication network between cells, molecular mediators, various signal pathways, and feedback loops that initiate and regulate the trauma-induced inflammatory response.

Exosomes are small extracellular vesicles (EVs) released by cells that play key roles in facilitating intercellular communication, both locally and systemically, under both homeostatic physiological and pathophysiological conditions [34,35,36,37,38,39,40,41,42,43]. These small (30–200 nm diameter) endosome-derived membrane vesicles are found in a variety of biofluids, and contain critical cellular messengers (e.g., cytokines, growth factors, RNA, transcription factors, adhesion molecules) [34,44,45,46,47,48]. Exosomes can affect target cells either by stimulating them directly via surface expressed ligands, or by transferring biologically active molecules between cells [34]. The cell types, insult and severity of injury, and cellular microenvironment influence the complex payload-content (cargo) of exosomes shed [34,45,49,50,51,52,53]. Both localized and distant exosomal-mediated delivery systems have been shown to be critical drivers in the initiation, progression, and resolution of inflammation [37,45,54,55,56,57]. Importantly, the cargo of circulating exosomes is now considered to contain important early molecular signatures and surrogate diagnostic markers of inflammation and disease states in multiple pathologies, especially cancer [35,36,37,54,58,59,60,61,62,63,64,65,66].

Here, we provide a brief overview of exosomes and the inflammatory response to trauma to establish a foundational base in order to identify research gaps and opportunities to bridge the two fields. Specifically, we highlight the role of exosomes in the response to severe trauma and discuss the possibility of bioactive molecules contained within exosomes to serve as biomarkers in the diagnosis and prognosis of trauma-associated inflammation and complications.

## 2. Biological Characteristics of Exosomes

### 2.1. Biogenesis and Intercellular Communication

Extracellular vesicles are small, non-replicative membrane-bound structures released from cells, which serve as carriers of biomolecular materials between cells, both locally and systemically. These vesicles can be isolated from a variety of biofluids, including blood, urine, saliva, breast milk, amniotic fluid, ascites, cerebrospinal fluid, bile, and semen [34]. There are several types of small circulating vesicles, including exosomes, microvesicles (also termed ectosomes), and apoptotic bodies. Aside from their function in biological processes, the major difference between these EV subpopulations is their intracellular origin. Exosomes are derived from endosomes, whereas microvesicles are formed by the budding of the plasma membrane, and apoptotic bodies are vesicles released into extracellular space via plasma membrane blebbing during the disassembly of dying cells [67,68,69]. The EV subtypes also vary in size, with overlapping diameter ranges, spanning from 30 to 200 nm for exosomes, 100 to 1000 nm for microvesicles, and 50 to 5000 nm for apoptotic bodies [44,45,46,70]. Microvesicles and exosomes function similarly in the transport of cargo biomolecules, and can be released under similar conditions, whereas apoptotic bodies are only released from dying cells [34]. Both microvesicles and exosomes are released constitutively and in response to cell stress, cellular activation, senescence, and pathological conditions; therefore size, origin of vesicle release, and composition are important in discriminating between these EVs [34,71,72,73].

**Figure 1:**
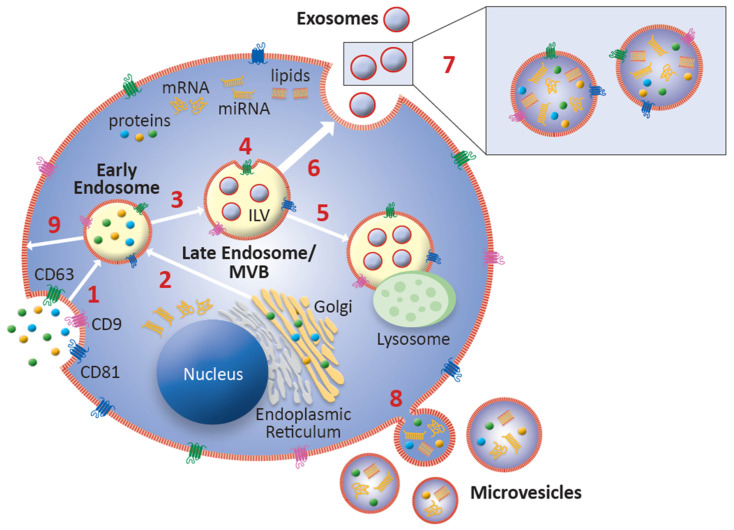
Exosome biogenesis. (1) Invagination of the plasma membrane of the cell collects extracellular material and generates a membrane bound endosome within the cell which retains plasma membrane proteins such as tetraspanins. (2) Golgi network packaging and transport of cellular material to the endosome. (3) Endosomal sorting complex required for transport (ESCRT) machinery packaging of biomaterial. (4) Invagination of the endosome forms intraluminal vesicles (ILVs). The collection of ILVs within an endosome forms a multivesicular body (MVB). There is cellular content from the cytosol contained within the ILVs as a result of microautophagy. (5) Trafficking of the MVB to the lysosome. MVB fusion with the lysosome results in ILV degradation. (6) Exosomes are released when the ILVs are released following MVB fusion with the plasma membrane. (7) Close-up of exosomes at release, with tetraspanins and bioactive cargo. (8) Budding of the plasma membrane forming microvesicles. These extracellular vesicles (EVs) are distinguishable from exosomes by nature of their biogenesis, and size. (9) Recycling of early endosome content to the plasma membrane.

Exosomes are generated from intraluminal vesicles (ILVs) within late endosomes, which are also known as multivesicular bodies (MVBs) (Figure 1). In brief, ILVs form via invagination of the endosome during a process known as microautophagy, therefore containing cell-specific cargo of proteins, lipids, and nucleic acids. A lipid bilayer, similar to a cell’s plasma membrane, makes up the outer membrane of exosomes [44]. The formation of ILVs and packaging of their contents can be Endosomal Sorting Complex Required for Transport (ESCRT)-dependent or ESCRT-independent [74,75]. MVBs are either trafficked to lysosomes for fusion and ILV content degradation, or to the plasma membrane, where ILVs are released as exosomes into the extracellular space via exocytosis upon fusion with the plasma membrane [71,76]. While several molecules have been identified in the trafficking of MVBs to the plasma membrane for exosome release, including the ESCRT proteins, the exact mechanism of this process is still unknown [74,77]. Proteins and genetic material from the cell of origin are delivered via exosomal binding to the plasma membrane of target cells, or by cellular phagocytosis of exosomes by target cells [34,37]. Uptake of exosomes by target cells is mediated by the interaction between exosomal ligands and receptors of recipient cells wherein they play a pivotal role in local and distant intercellular communications in modulating and coordinating the cellular functional activities of adjacent target cells, as well as those in distant tissues and organs [78]. The lipid bilayer of exosomes makes them unique cellular communication vehicles. Exosomes are stable in circulation and are able to shield their cargo from the microenvironment, leading to stability of the bioactive material within, and increased efficiency of delivery to effector target cell [64,79,80,81,82].

### 2.2. Exosomal Cargo Overview

Exosomal cargo is dependent on the cell of origin and its state of activation [75,83,84]. Exosomes contain a variety of bioactive molecules, to include cytosolic proteins (e.g., cytokines, growth factors), integrins, vesicle transport associated molecules, cytoskeletal proteins, heat shock proteins (HSPs), enzymes, lipids, signaling molecules, RNAs, and glycosylated transmembrane scaffold proteins called tetraspanins which mediate diverse functions including cell motility, protein trafficking, cell adhesion, and intra-cell signaling coordinating ligand-receptor interactions [46,47,48,77]. Exosomal cargo contains both proteins and their mRNA transcripts, suggesting a redundancy in the loading of exosomes, as well as the importance of their cargo [85].

The nature of the biogenesis of exosomes results in variations in protein content based on the parent cell and the sorting mechanism. Proteomic analysis has revealed several different types of protein modifications involved in the processing of proteins for packaging into exosomes, including phosphorylation, ubiquitination, ISGylation, SUMOylation, myristoylation, glycosylation, and oxidation [44]. Tetraspanins have emerged as a unique protein identifier of exosomes in enrichment and isolation processes. Further, tetraspanins may have roles in exosome biogenesis, packaging of cargo, binding and uptake by target cells, and exosomal antigen-presenting functions in the immune response [86]. CD63, considered a specific marker for exosomes, indicates endosomal origin and is involved in the formation of ILVs [86]. CD63 is found in late endosomes and contains an internalization motif that promotes rapid endocytosis. CD81 and CD9 are structurally similar surface membrane proteins with broad distribution on diverse cell types where they play a critical role in cell–cell fusion, especially in macrophages [87]. EVs containing both CD63 and CD81 tetraspanin proteins are phenotypically classified as exosomes. The identification of exosomal tetraspanins can vary based on the parent cell, and various combinations of the markers CD9, CD63, and CD81 indicate successful exosomal isolation in the laboratory [86].

Two main structural forms of RNA are found in exosomes: coding RNAs like messenger RNA (mRNA), and non-coding RNAs, which include both micro-RNA (miRNA) and long non-coding RNA (lncRNA). The mRNA found in exosomes is functional, and able to be translated to proteins when exosomes are taken up by target cells [85]. However, size analysis of these mRNAs shows they are between the length of miRNAs and full-length mRNAs, suggesting that exosomes carry primarily truncated, but functional, post-transcriptional mRNAs [88]. miRNAs are short (20–25 nucleotides) non-coding RNAs that participate in post-transcriptional modifications, specifically with the end result of silencing target mRNAs. This is accomplished either by complementary binding with target mRNAs causing decreased expression of proteins, or by partial binding resulting in mRNA degradation. Thus, the effects of changes in miRNA expression can be observed both in terms of target mRNA levels or in target protein output [89]. The cleavage of pre-miRNAs by the Dicer endonuclease also produces antisense miR* passenger strands, which are less stable than their mature miRNA counter-parts, but may be biologically active [90]. These antisense miRNAs can also be found in exosomes [91,92]. miRNAs are differentially expressed across cell and tissue types, and their loading into exosomes is reflective of cellular activation, making them a valuable target for monitoring of disease [93,94,95,96]. lncRNAs include a diverse and dynamic population of non-coding RNAs longer than 200 nucleotides and can be a source of miRNA biogenesis [97,98]. Similar to miRNAs, lncRNAs modulate protein expression via post-transcriptional mRNA modifications, but are also able to interfere with mRNA transcription, and modulate expression of other noncoding RNAs [99,100]. Given the diversity of exosomal content, the isolation of exosomes and analysis of their cargo may provide critical insights to the development and progression of various disease states.

## 3. Inflammation and the Role of Exosomal Cargo

### 3.1. Inflammatory Response to Severe Trauma

**Figure 2:**
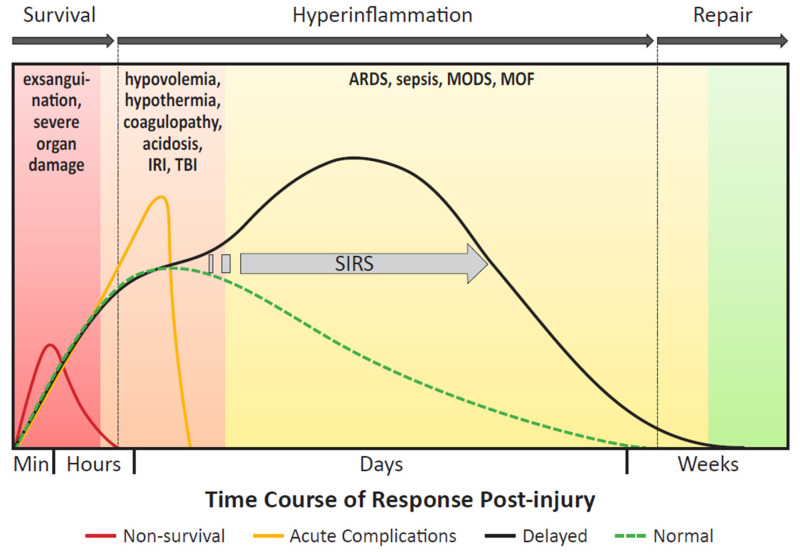
Response to traumatic injury. The three phases following injury are identified by the arrows at the top of the timeline: survival, hyperinflammation, and repair. The longer the hyperinflammation phase is extended, the more delayed the initiation of the repair phase, contributing to late term healing complications. The text beneath the response phase arrows lists various complications associated with increased mortality during the course of the body’s response to trauma—the majority of these conditions after the survival phase are caused by or related to inflammation. The dotted green line shows a “normal” appropriate, restrained inflammatory response that begins to resolve a few days after injury. The red curve represents non-survival—patients that will expire immediately following injury or within the earliest phases of care due to insurmountable injuries and exsanguination. These patients will fail to survive long enough to amount a robust inflammatory response. The orange curve represents patients that will be lost in the first few days of care, usually due to complications related to resuscitation, including ischemic reperfusion injury (IRI), or injury severity, especially in the setting of traumatic brain injury (TBI). The black curve encompasses the patient population that will experience delayed trauma-associated hyperinflammation-induced complications, beginning several days after the initial injury. Dysregulation of the inflammatory response or further insults will cause a patient to deviate from normal recovery, making them more susceptible to the development of systemic inflammatory response syndrome (SIRS), or infection that may cause further complications or death.

The body’s response to severe traumatic injury can be characterized by three acute phases: survival, hyperinflammation, and repair (Figure 2). Death after traumatic injury is classically described in accordance with a trimodal mortality model, wherein death occurs immediately (< 1 h), early (< 24 h), and late (> 24 h) following injury [101]. Patients who sustain insurmountable injuries to major organ systems, including catastrophic hemorrhage from great vessel disruption or severe intra-cranial injuries, will typically die during the survival phase in the immediate to early stages of care [102,103]. Patients that initially survive often face severe hypercoagulable, hyperinflammatory, and infectious complications, principally during the inflammatory phase of the acute response. Patients who sustain polytraumatic injuries have a greater risk of developing these critical acute complications, as they experience a more prolonged and robust inflammatory response than patients sustaining a singular injury. As a result of a prolonged and heightened inflammation phase, the repair phase is ultimately delayed, as it cannot be initiated until the successful resolution of hyperinflammation. Over-activation of the immune system also interferes with other components of the response to severe trauma, including hemostasis and anti-microbial defenses [5,104,105]. Importantly, an exaggerated and lengthy hyperinflammatory response can increase the risk of developing tissue repair complications, MODS, MOF, sepsis, and death.

The underlying pathophysiology of trauma-induced inflammation remains elusive, and the mechanisms are complex and multifactorial. Identification of early biological signatures to predict development, progression, and clinical outcome of severe inflammation-mediated immune dysfunction is critical for timely intervention and mitigation of downstream effects. Prior research efforts have largely focused on individual or small numbers of classic systemic inflammatory markers as biological signatures [4,106,107,108,109,110,111,112,113,114,115]. A combinatorial signature involving genome-wide multi-plexed analyses of interlinked pathways may have a greater predictive value at any given point. Importantly, much of the underlying inciting biomolecular signaling that contributes to early immune dysregulation may be overlapping and/or distinct in the activation of the innate immune system preceding the development of a measurable systemic response. Additionally, the use of exosome-associated biomarkers found in circulation may serve as an early stage, non-invasive “liquid biopsy” companion to facilitate real-time diagnosis and monitor clinical progression, recovery, pathological complications, and therapeutic responses.

The innate immune system is activated within minutes after acute injury [5,26]. Following trauma, there is an uncontrolled local and systemic release of endogenous mediators into the circulation as a result of tissue damage and cellular necrosis associated with hemorrhage, ischemia-reperfusion injury (IRI), musculoskeletal injuries, burns, surgical procedures, subsequent secondary tissue injuries, and other insults [30,52,116,117,118,119]. These tissue-derived DAMPs include high-mobility group box 1 (HMGB1), the S100 proteins, HSPs, and mitochondrial DAMPs (mtDAMPs) [28,29,120,121]. In severe trauma, HMGB1, a DNA binding protein and key amplifier of inflammation, is actively secreted by immune inflammatory cells and passively released by injured and necrotic into the extracellular microenvironment to modulate the activation, metabolic activity and migration of immunocompetent cells and other cells at the site of injury [110,122,123,124].The S100 proteins participate in calcium homeostasis intracellularly and are passively released in the case of cell necrosis, but also play a role in cytokine release and influencing cell migration in inflammation [120,125]. While HSPs normally provide functions as chaperone proteins, they are also stress responsive proteins upregulated in traumatized tissues [126]. Similar to the other DAMPs, mtDAMPs are released by cells in response to damage, particularly mechanical and oxidative trauma. mtDAMPs participate in the trauma-induced immune response, and their activity has also been linked to specific injury patterns, including fracture [121,127,128]. Invading pathogens contain biochemical signatures known as PAMPs, with the most recognized being endotoxin, also known as bacterial lipopolysaccharide (LPS), found on Gram negative bacteria. The innate immune system senses the diverse biochemical signatures of DAMPs and PAMPs via pattern recognition receptors (PRRs), including extracellular Toll-like receptors (TLRs), cytosolic nucleotide oligomerization domain (NOD)-like receptors (NLRs), and the receptor for advanced glycation end products (RAGE), allowing these danger signals to contribute to inflammation by activating and recruiting immune cells.

Detection of DAMPs and PAMPs propagates a cascade of early innate intracellular signaling, leading to the activation of key transcription factors, such as nuclear factor kappa beta (NF-κB), that regulate diverse cellular responses and the transcription of pro-inflammatory mediators to include cytokines, chemokines, interferons, and anti-microbial peptides [28,29,121,129]. Endothelial activation, and local inflammatory mediators like histamine, kinins, and arachidonic acid cause leaky barriers and edema, allowing extravasation of neutrophils, monocytes, and macrophages into the tissue [4,32]. Monocytes are recruited from the circulation or the splenic reservoir to the site of inflammation where they infiltrate the tissue and undergo marked phenotypic and functional differentiation into macrophages or dendritic cells (DCs) to join and/or replace tissue-resident macrophages, all of which have been shown to play distinct roles in resolving inflammatory responses and promoting tissue repair-regeneration. [130,131,132,133]. Macrophage polarization is a key part of this process, explained by the M1/M2 paradigm. The M1 phenotype is pro-inflammatory, while the M2 phenotype is pro-reparative and healing [130,134]. Classically activated M1 macrophages activated by DAMPs or in response to pathogens release a milieu of early pro-inflammatory mediators (interleukin 1 beta (IL-1β), IL-6, IL-8, IL-12, tumor necrosis factor alpha (TNF-α), macrophage inflammatory protein 2 alpha (MIP-2α/ CXCL2), interferon gamma-induced protein 10 (IP-10/CXCL10), regulated on activation, normal T-cell expressed and secreted (RANTES/CCL5), reactive oxygen species (ROS), and reactive nitrogen species (RNS)). At the site of inflammation, these mediators propagate the pro-inflammatory cascade by activating endothelial cells and inducing the recruitment of other immune cells into the inflamed tissue by activating more macrophages and inducing sustained inflammatory responses [28].

The binding of DAMPs to PRRs on innate immune cells triggers both intense pro-inflammatory, and concomitant anti-inflammatory responses. The initial anti-inflammatory response plays a critical role in balancing the hyperactive inflammatory state in the setting of excessive injury to the host following severe trauma [135]. However, both uncontrolled pro- and anti-inflammatory responses may lead to pathological consequences. While excessive inflammation promotes the development and propagation of secondary tissue injuries beyond the initial site of injury/damage, the anti-inflammatory response can often lead to host defense impairment and sepsis, frequently leading to severe complications, MODS, and death. DAMPs and PAMPs bind similar cellular receptors, and post-traumatic cellular stimulation with DAMPs can lead to receptor tolerance upon pathogen exposure, such that cells fail to mount an appropriate response when binding to pathogen-triggered PAMPs, leading to infection [136]. This impaired infection response is compounded in a state of systemic inflammation by reduced human leukocyte antigen-DR expression (HLA-DR) on peripheral blood mononuclear cells, which may pre-dispose patients to sepsis [103].

**Figure 3:**
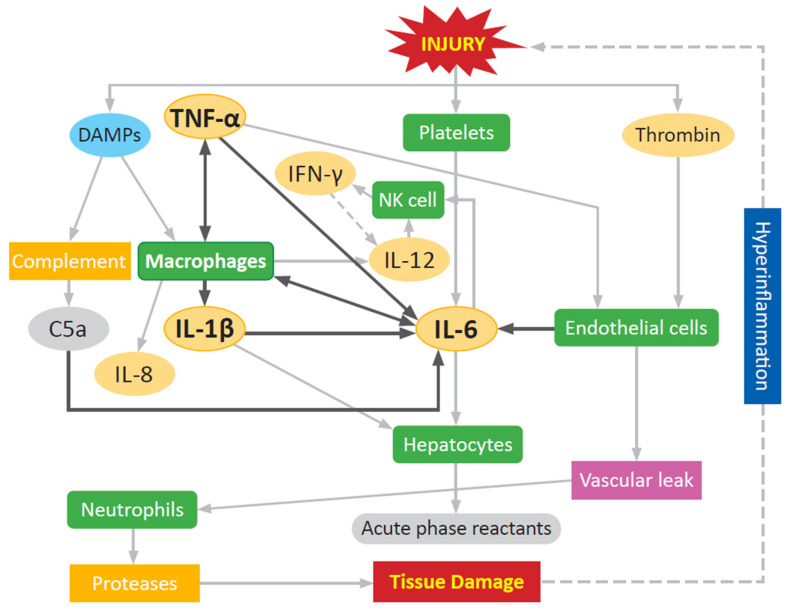
Inflammatory cytokines and the self-propagating trauma cycle. This diagram is a simplified representation of the complex web of pro-inflammatory cytokines and how continued activation leads to further tissue damage. The release of damage associated molecular patterns (DAMPs), platelets, and thrombin in response to injury activates macrophages and endothelial cells to mount a response. Cytokines, including interleukins (IL), and tumor necrosis factor (TNF)-α are key early messengers in the inflammatory response, acting to activate natural killer (NK) cells, macrophages, neutrophils, and hepatocytes. Hyperinflammation from an unrestrained response further contributes to tissue damage, engaging the inflammatory response again.

Regulation of innate immune cell mediated pro-inflammatory response at sites of tissue injury/damage is orchestrated through the early release of the pro-inflammatory cytokines IL-1β, IL-6, and TNF-α (Figure 3). The development and severity of an inflammatory response can be monitored by tracking systemic levels of IL-1β, IL-6, IL-8, TNF-α, and MIP-1α. High serum levels of these mediators is indicative of the body being in a hyper-inflammatory state [5,114]. This array of soluble mediators directly evokes further immune cell activation in different cell types through the stimulated synthesis and release of a plethora of cytokines, chemokines, and other pro-inflammatory mediators used to communicate with other cells that initiate and constrain inflammatory response to pathogens and injury. These highly regulated, sequentially, and temporally orchestrated pleiotropic proteins initiate a cascade of signaling events that regulate host defenses against pathogens, leukocyte chemotaxis and migration, vascular endothelial permeability (vascular leak), production of acute phase proteins, and tissue damage repair. TNF-α is one of the first cytokines released following injury, peaking 1–2 h after injury, with a short serum half-life of about 20 min [4,115]. TNF-α contributes to endothelial activation and vascular leak while also stimulating natural killer (NK) cells, monocytes, and macrophages. IL-1β induces the production of cytokines (IL-6 and TNF-α), and chemokines (monocyte chemoattractant protein 1 (MCP-1)/CCL2 and MIP-1α/CCL3) from immune cells, and the production of acute phase proteins including C-reactive protein (CRP) from the liver [4,137]. IL-1β has a longer half-life at 90 min, but this is still a small window in the setting of acute trauma care, making fluctuations of the cytokine itself more difficult to monitor, and pointing towards downstream effectors as signs of activity [4,138]. IL-1β is critical for the acute development of inflammation, and inability to produce IL-1β results in an impaired systemic inflammatory response to stimuli, likely due to the resulting failed induction of IL-6 [139]. Without IL-6, the immune system is unable to mount a normal inflammatory response [139]. Both TNF-α and IL-1β promote the production of IL-6 by macrophages. IL-6 activates NK cells, inhibits apoptosis of neutrophils, and mediates the release of acute phase proteins from hepatocytes [4,140]. The interlinked pathways and redundancy of pro-inflammatory activation between TNF-α, IL-1β, and IL-6 allows for general monitoring of inflammation using any one of the cytokines as a reference, instead of each separately [141]. Prolonged and elevated increases in circulating levels of IL-6 post-injury correlates with a high Injury Severity Score (ISS), and is a reliable predictor of MODS, MOF, and mortality in polytrauma patients [111,115]. Levels of IL-6 can peak as early as four hours post-injury in major trauma, and can estimate trauma severity up to six hours post-injury [113]. However, the predictive value of IL-6 for major secondary complications like ARDS and MODS, and mortality peaks around 72 h [114]. The half-life of IL-6 is longer than TNF-α and IL-1β, around 15 h, making it easier to monitor in serum or plasma using commercially available immunoassay detection platforms [142]. This makes IL-6 a more reliable indicator of the progress of inflammation and immune response, because while TNF-α can induce SIRS on its own and IL-6 cannot, the early window of release and short half-life make TNF-α an unsuitable marker. Circulating IL-6 levels often serve as strong predictors to guide escalation of treatment in patients with high risk of developing hyperinflammatory syndrome [4,115]. IL-8 (CXCL8) is a major inflammatory chemokine released early post-injury by macrophages, at which point it plays a key role in the recruitment of neutrophils and other immune cells to the site of injury [4,140]. In addition, IL-8 plays a pivotal role in other wound healing processes including keratinocyte/fibroblast proliferation, angiogenesis, and tissue remodeling [143,144]. It can remain persistently elevated following trauma, up to a period of weeks after the initial inflammatory response [4,115]. Elevations of IL-8 are correlated with patients developing ARDS and MOF, as well as increased mortality when significantly elevated at 24 h. This is due to the chemotactic function of the cytokine, attracting neutrophils to organs where they then exhibit destructive effects through degranulation and the release of nitric oxide (NO) and ROS [23,115]. Prolonged inflammation increases the time of neutrophil extravasation, and this increases damage to the tissue, resulting in a self-propagating cycle of tissue destruction and response, and delaying wound healing and tissue repair (Figure 3) [5,19].

Severe trauma not only induces pro-inflammatory reactions, but also anti-inflammatory reactions to reverse this process via CARS. Once the inflammatory response is established, control is critical. The inability of IL-4, IL-10, and other anti-inflammatory cytokines like tumor growth factor beta (TGF-β) to deactivate the inflammatory cascade results in a transition to a persistent inflammatory state. IL-10 is a cytokine that signals for attenuation of the immune response [5,114]. This is a rapid response, with IL-10 being released within 60 min of trauma [115]. In SIRS, high levels of IL-10 are indicative of the body attempting and failing to control an inflammatory response [5]. The interplay of IL-6 and IL-10 is an indicator of patient prognosis. In the final peak of the trimodal polytrauma mortality model, patients with a low ratio of IL-6 to IL-10 24 to 72 h after injury have a higher incidence of ARDS, MODS, and death (Figure 2) [114]. TGF-β works to dampen the immune response by inhibiting pro-inflammatory cytokine production and suppressing macrophages, DCs, and NK cells, in addition to its effects on the adaptive immune response [145].

The innate immune system, while providing the robust inflammatory response to trauma, is not the only responder. While the innate immune response provides a rapid and non-specific response, adaptive immunity is delayed, specific, cell-mediated, and humoral. Similar to the response of the innate immune system, the adaptive immune response can be a double-edged sword—necessary to clear dead, necrotic tissue and pathogens, but with the potential to become dysregulated and potentiate systemic inflammation and further tissue damage. While SIRS is primarily mediated via the innate immune response, CARS is mediated by the adaptive immune response [146]. Briefly, the adaptive immune system begins to respond days following initial insult, as opposed to the minutes it takes to activate the innate immune response, with DCs acting as a conduit between the two systems to control T-cell responses, specifically that of CD4^+^ T cells in inflammation. DCs are antigen presenting cells that act as sentinels, sensing the presence of microbes and tissue damage. Outside of sentinel functions, DCs can be matured by activated NK cells. Together, these cell types engage in immunoregulatory crosstalk via cytokines and cell-to-cell contact [147,148]. Once activated, DCs induce naïve CD4+ T cell activation and proliferation, including differentiation into T helper (Th) subsets Th1, Th2, and Th17, as well as T regulatory (Treg) phenotypes. The inflammatory Th1 cells produce interferon gamma (IFN-γ), which results in classical activation of M1 macrophages and the production of TNF-α, while anti-inflammatory Th2 cells produce IL-4, IL-5, and IL-13 causing alternative M2 macrophage activation [134,149]. Th17 production of IL-17 aids in neutrophil recruitment, a key aspect of the response to infection [150]. While infection stimulates Th1 and Th17 responses, trauma is known to suppress CD4^+^ T cell responses [151]. Studies have reported both the elevation of levels of Th2 associated cytokines leading to decreased survival in trauma patients with sepsis and decreases in both Th1 and Th2 responses following trauma, pointing to the importance of Th1 and Th2 balance in effective injury response [135]. Tregs are responsible for restraining the activity of the Th subtypes mediated primarily via IL-10 and TGF-β [152]. Injury-induced activation of Tregs appears to be responsible for controlling the CD4^+^ T cell response in trauma, with Treg depleted mice showing appropriate Th1 inflammatory response following injury [153]. The presence of high levels of IL-10 in SIRS is linked to CARS, with IL-10 being a powerful immunosuppressive cytokine produced by multiple adaptive immune cells, including DCs, Th2, and Treg cells [146,152,154,155]. This is consistent with CARS being associated with increased Th2 and Treg activity [151]. Appropriate engagement of the adaptive immune system is necessary for a balanced immune response, but escalation to CARS, like SIRS, can be detrimental.

### 3.2. Role of Exosomes in Regulating Inflammation

Damage associated molecular patterns contained within exosomes have been shown to activate the innate immune system, and therefore have the potential to contribute to the development of SIRS following traumatic injury [51,156]. While not yet identified specifically in exosomes isolated from patients during the early phase after severe trauma, many DAMPs are locally and systemically detectable following several different injury patterns, including fracture, hemorrhage, burn, and IRI [30,52,116,117,118,119]. Circulating DAMPs of interest in the study of trauma and inflammatory disease that have been identified in exosomes are HMGB1, the S100 proteins, HSPs, and mtDAMPs [51,85,120,157,158,159,160,161,162,163]. Physiologic stressors can modulate the location of HSPs in relation to exosomes, with acute stress increasing Hsp72 expression on plasma exosomes, and decreasing intra-vesicular miRNAs miR-142 and miR-203 [51,164].

Macrophages release exosomes that activate, and induce pro-inflammatory responses in naïve immune cells, including differentiation of mononuclear cells into active macrophages releasing pro-inflammatory cytokines [54,165]. M1 macrophage-derived exosomes containing miR-155 are able to regulate macrophage polarization, decreasing the anti-inflammatory M2 phenotype [166]. Through TLRs, DAMPs, PAMPs, and other inflammatory signaling increases miR-155 expression in macrophages [166,167,168]. Upregulation of miR-155 increases inflammatory cytokines including IL-6, IL-1β, TNF-α, and IL-8, and decreases anti-inflammatory cytokines, namely IL-10, and TGF-β [166,167]. Exosomes also carry inflammasome proteins, which are necessary to generate the biologically active form of IL-1β, a key cytokine in immune system activation responsible for fever and leukocyte recruitment [49,169,170]. Exosomes derived from NK cells contain cytotoxic proteins and are able to mediate cytotoxic functions similar to their donor NK cells [171]. Once the inflammatory response is established, exosomes can propagate inflammation and impair healing, as well as inducing production of inflammatory cytokines and chemokines [172,173]. Macrophages co-cultured with mesenchymal stromal cell (MSC)-derived exosomes exhibit higher rates of oxygen consumption, and translocation of NF-κB, a key component of cellular regulation of inflammatory factor transcription [174]. Differentially expressed proteins in exosomes under inflammatory conditions are linked to other components of the physiologic response to trauma including the complement and coagulation cascades, such as complement C3, complement C4b, fibrinogen gamma chain, fibrinogen beta chain, and plasminogen activator inhibitor 1 [54,175].

Exosomes post-injury contain important regulatory cytokines, growth factors, repair mediators and functional RNAs, such as mRNA and miRNA, that play important roles in inflammation and coordinating wound repair-healing [176]. A deficiency in exosome secretion results in a persistent inflammatory phenotype and inappropriate response to inflammatory stimuli [55]. TGFβ-1 is an important activator of MSCs, which play a key role in the healing and repair process. Exosome associated TGFβ-1 exerts a more potent effect on MSC migration and phenotype than the freely circulating cytokine [177]. miR-146 is an anti-inflammatory mediator and associated with the NF-κB signaling pathway [168,178,179,180]. IL-1β stimulates production of miR-146, and its packing into exosomes. Exosomal miR-146 has been reported to enhance M2 polarization of macrophages [181]. Within the miR-146 family, miR-146a targets TNF receptor associated factor (TRAF) 6, IL receptor associated kinase (IRAK) 1, and IRAK2, resulting in negative regulation of IL-8 and CCL5 [182]. miR-146a has both pro- and anti-inflammatory effects, whereas miR-146b is only anti-inflammatory [183]. Myeloid-derived suppressor cell (MDSC)-derived exosomes, and miR-126 from endothelial-derived exosomes also promote M2 polarization [163,184]. Given the role of miRNAs in modifying cytokine expression, changes in miRNA expression and transport mediated by exosomes in response to acute trauma could provide insights into subtle changes in the innate response that may potentially lead to systemically devastating inflammatory cascades, prior to clinical detection of IL-6. The ability to load miRNAs into exosomes makes them a unique delivery vehicle, especially in the context of combating an inflammatory condition like sepsis with an anti-inflammatory miRNA like miR-146 [185].

Research regarding the mechanistic role of exosomal lncRNAs in the innate immune inflammatory response to injury and infection is limited, as many studies have focused on the utility of exosomal lncRNAs as disease biomarkers [66]. Many immunomodulatory lncRNAs have been identified in exosomes derived from cancer cells, evidenced by their frequent tumor-associated naming conventions, but must be interpreted in the context of the unique tumor microenvironment [186,187,188]. In a murine model of acute lung injury, the reduction of the lncRNA termed colorectal liver metastasis-associated transcript 3 (CLMAT3) found in monocyte-derived exosomes was reported to upregulate NF-κB activation, resulting in increased IL-1β, IL-6, and TNF-α production that contributed to pathogenesis [189]. In a controlled cortical impact traumatic brain injury model, Patel et al. demonstrated the immunomodulatory impact of therapeutic exosomal delivery of lncRNAs. Infusion of human adipose-derived stem cells (hASC)-derived exosomes containing the lncRNA termed metastasis-associated lung adenocarcinoma transcript 1 (MALAT1) modulated both coding and non-coding inflammatory RNAs, reduced neural lesion volume, and attenuated behavioral and motor symptoms, indicating importance of future studies for development of lncRNA exosome-based diagnostic and therapeutic tools [100].

Exosomes also play a role in the response of the adaptive immune system. DC-derived exosomes exchange major histocompatibility complex molecules between DCs and activate naïve CD4^+^ T-cells [190,191]. DC-derived exosomes also participate in the transfer of miR-155 and 146a, key immunomodulatory miRNAs [185]. Bystander DCs undergo maturation when bound by DC-derived exosomes presenting TLR-ligands, amplifying the pro-inflammatory response through increased Th1 polarization [148]. T cell receptor triggering increases the number of exosomes released from CD4^+^ T cells [192]. Exosomal Hsp70 from DCs is able to modulate T cell differentiation [193]. “Lethal” exosomes containing membrane-Fas Ligand released from T lymphocytes are able to trigger Fas-dependent apoptosis, a key mechanism of immune homeostasis in controlling over-active or auto-reactive T cells [194].

The cargo contained in exosomes has important functions in controlling immune cell function acting across all aspects of the inflammatory response. The extracellular communication system has been investigated more in depth in cancer and the tumor environment but is critical across a multitude of pathologies. Identifying and understanding these roles will not only aid in the comprehensive understanding of how the body reacts to trauma, but also may serve as early indicators for immune dysregulation leading to delayed complications following traumatic injury.

## 4. Exosomes and Their Cargo in Trauma-Associated Complications

The delayed effects of trauma manifest days to weeks post-injury. These issues can be compounded by interventions such as aggressive fluid resuscitation and surgery that further stress the body and contribute to inflammation. Identifying early signs of immune dysfunction following acute injury is critical for intervention and prevention of clinical sequelae. While the clinical symptoms can develop rapidly, activation of the innate immune system and the inflammatory response can potentially be identified on a biomolecular level preceding the development of a systemic response. Multifactorial, yet poorly defined, cellular, genomic, and proteomic inflammatory immune responses in patients with severe trauma, severe burns, or bacterial infection are well-documented [19,195,196]. These characterizations have increased understanding of the physiologic response to trauma; however, further investigation is necessary for how these changes correlate with injury, and the timeline of response to injury. Current characterizations have been performed on samples taken several hours to days after injury, demonstrating the prolonged inflammatory impact, but not capturing the inciting biomolecular signaling. The ability of exosomal cargo such as miRNAs to modify expression of proteins in target cells and manipulate cytokine production make them valuable potential targets for early identification of inflammatory complications. In this section, we examine the consequences of trauma-associated inflammatory complications and the exosomal cargo identified in the setting of their development, including IRI, ARDS, and sepsis, as well as highlighting the potential for future investigation into MODS and MOF (Table 1).

### 4.1. Resuscitation and Ischemia-Reperfusion Injury

Polytrauma frequently involves major vessel disruption and hemorrhage, which can rapidly develop into hemorrhagic/hypovolemic shock. Volume loss and shock-induced vasoconstriction leads to insufficient oxygen delivery to cells throughout the body and ischemic tissue injury. This ischemic environment is locally worsened in distal extremities when prolonged tourniquet use is necessary to prevent exsanguination [119,215]. Fluid resuscitation, whether by blood or other substitutes, is necessary to improve oxygen delivery [216]. However, while this fluid resuscitation can improve initial survival, it was noted in the Vietnam War that overly aggressive fluid resuscitation resulted in increased deaths due to ARDS as a late complication [217]. Early endothelial damage from reperfusion results in leaky barriers and increased leukocyte extravasation [216].

Ischemia-reperfusion injury is a unique injury pattern characterized by a period of hypoxia, followed by sudden hyperoxia, gradually returning to the normoxic state. Tissue damage is induced by cellular ATP depletion and an intense pro-inflammatory process. The latter is initiated by the ischemic period (hypoxia), but occurs mainly during the reperfusion phase, and is characterized by a large neutrophil recruitment (innate immune response). NF-κB is a redox sensitive translational factor, which upregulates TNF-α production, initiating the pro-inflammatory cytokine cascade, including production of IL-1, IL-6, and IL-8 [218]. This inflammatory response is an appropriate reaction to ischemia-induced cellular necrosis. Upon reperfusion however, neutrophils (innate immune response) and CD4^+^ T cells (adaptive immune response) are recruited to the site of injury, amplifying the inflammatory response, resulting in accumulation of free radicals, thereby potentiating systemic effects of local IRI [219]. The local inflammatory response as a result of IRI includes the simultaneous production/release of cytokines (TNF-α, IFN-γ, IL-1, IL-6), chemokines (IL-8), danger signals, ROS, and other mediators resulting in complex aberrant activation of multiple signaling pathways and increased apoptosis, leading to the temporary or permanent functional impairment of an organ or tissue, having implications either locally or at a distant site. IRI can lead to serious clinical manifestations to include SIRS, MODS/MOF, extremity amputations, and even death.

Kojima et al. used a rat model of trauma and hemorrhagic shock to demonstrate modulations in the concentration of circulating exosomes in mesenteric lymph during normostasis, hemorrhagic shock, and in the resuscitation phase [220]. The concentration of exosomes increased during shock and decreased during resuscitation. While the cargo of these exosomes was not profiled, the exosomes isolated during the resuscitation phase induced higher levels of monocyte NF-κB expression and increased intra-cellular TNF-α levels in macrophages [220].

Hypoxic conditions and the induction of HIF-1α increases the release of exosomes from various cell types, including renal proximal tubular cells, and cardiomyocytes [221,222]. Cancer cells under hypoxic conditions are known to release exosomes with increased miR-23a, but Crouser et al. recently identified elevated exosomal miR-23a in patients with myocardial ischemia [197,223,224]. miR-23a impairs NK cell cytotoxicity and is associated with TGF-β and angiogenesis in the tumor microenvironment [223,224]. Knockdown of miR-23a causes suppression of the NF-κB pathway, and results in downstream reduction of IL-6 and TNF-α production [225]. Exosomes released from cardiomyocytes cultured under hypoxic conditions contain functional TNF-α and induce apoptosis of other cardiomyocytes [222]. The mRNA cargo in exosomes released from cells under oxidative stress is significantly different than the exosomal cargo from untreated cells and is able to confer oxidative stress tolerance to other cells [202]. Eldh et al. reported 40 differentially expressed mRNA transcripts in exosomes derived from cells undergoing oxidative stress. The most upregulated was Vsig1, which codes for a cell adhesion protein, and the most downregulated was Ctnna1, which codes for beta-catenin, an adherens junction protein [202]. Circulating serum exosomes in a model of skeletal muscle IRI induced by clamping of the femoral artery contained increased levels of complement component C3 prepropeptide, PK-120 precursor, alpha-amylase one precursor, beta-enolase isoform 1, and adenylosuccinate synthetase isozyme 1 [203]. In another model of skeletal IRI induced by tourniquet, plasma exosomes contained significantly upregulated levels of miR-24 compared to sham. When these exosomes were added to cells cultured under oxidative stress, the transfer of miR-24 resulted in reduced apoptosis and decreased Bim expression in these cells, suggesting a protective role for the cargo of IRI-induced exosomes [198]. Zheng et al. showed that DC-derived exosomal delivery of Hsp70 to naïve T cells stimulated the PI3K/mTOR axis leading to increased Treg differentiation and decreased Th17 differentiation in a model of hepatocyte IRI [193].

Emanueli et al. found increases in five exosomal miRNAs (miR-1, miR-133a, miR-24, miR-210 and miR-133b) induced by ischemic injury following human coronary-artery-bypass-graft (CABG) surgery. These miRNAs positively correlated with cardiac troponin levels, but the appearance of elevated concentrations of circulating plasma exosomes appeared earlier than troponin, a gold standard in the clinical identification of cardiac injury and ischemia, particularly in cases of myocardial infarction. Increases in circulating exosomes were seen immediately post-operatively, whereas troponin elevations were not detectable until 24–48 h post-operatively [199]. In a mouse model of acute myocardial infarction, Cheng et al. demonstrated that plasma exosomes containing miR-1, miR-208, miR-499, and miR-133 were preferentially taken up by mononuclear cells within the bone marrow compartment within 6 to 12 h post-injury. The net effect was mobilization of bone marrow hematopoietic stem/progenitor cells with endogenous repair functions from the bone marrow to the site of ischemic injury [201]. Sonoda et al., using a model of kidney IRI induced by clamping the renal arteries, showed that urinary exosomal cargo could act as a biomarker of acute kidney injury progression. Urinary exosomes contained unique differentially expressed miRNAs in the injury phase (post-injury days 1 and 2), in the initial recovery phase (day 3–7), and in the extended recovery phase (day 7–14), allowing for discrimination between IRI and sham groups after injury resolution. One day after IRI, exosomal miRNAs with targets related to decreasing NF-κB activity (miR-16-5p, miR-24-3p, and miR-200c-3p) were increased [200,226,227,228]. On day 3, miRNAs with targets related to TGF-β signaling (miR-9a-5p, miR-141-3p, miR-200a-3p, miR-200c-3p, miR-429) were increased, with active TGF-β signaling manipulating the expression profile of these miRNAs in the extended recovery phase [200].

### 4.2. Systemic Inflammatory Response Syndrome

The onset of SIRS has been used a surrogate predictor of outcome in polytrauma. Clinical assessments such as ISS correlate a higher degree of polytraumatic injury with the onset of SIRS [229]. The clinical diagnosis of SIRS is based on changes in vital signs (temperature, heart rate, blood pressure, and respiratory rate) accompanied by a laboratory assessment of a change in blood leukocytes (leukocytosis, leukopenia, or bandemia) [230]. These inflammatory changes can also be seen post-operatively in patients undergoing major surgery [231,232]. Studies of trauma patients have created a “two-hit” model in correlation with IL-6 levels, where major reconstructive procedures or continued under-resuscitation in the early hyper-inflammatory post-trauma period causes patients to develop dangerously high levels of inflammatory cytokines, predisposing them to SIRS [111]. Systemic inflammatory mediators are monitored as a metric for tracking patient progress following severe injury, and progression to SIRS and other delayed complications [107,116,196,233]. Patient development of SIRS is a clinical indication of immune dysregulation, and a warning of potential patient decompensation and development of other inflammation-related complications, which have likely already been set into motion on a molecular level, including exosomal intracellular communication.

There is a lack of literature regarding the specific role of exosomes and their cargo in the development and resolution of SIRS. Closely related to SIRS is sepsis, and exosomes and their cargo have been more extensively investigated in that setting. While the role of exosomal cargo in immune communication, onset, and resolution has been investigated, and patient development of SIRS is directly related to immune control, exosomal changes have not been studied. Future investigation of how exosomes and their cargo relate to ISS and SIRS offer the potential for earlier identification of at-risk patients and may unravel a biomarker better suited clinically than the most commonly utilized current marker, IL-6, which often follows clinical signs that the patient is decompensating.

### 4.3. Acute Respiratory Distress Syndrome

Acute respiratory distress syndrome is a complex pulmonary state of acute lung injury (ALI) that can result in pulmonary edema, lung fibrosis, pulmonary hypertension, and catastrophic respiratory failure, with the leading cause of death being development of MOF. In the setting of trauma, the immune response, resulting in overwhelming systemic inflammation such as in SIRS, causes NF-κB activation and upregulation of pro-inflammatory mediators. This leads to the recruitment of neutrophils which release ROS and proteases that inappropriately damage the alveoli. Histological analysis of lung tissue shows hyaline membranes with diffuse neutrophil and macrophage invasion, protein-rich fluid in the alveolar spaces, disruption of the alveolar epithelium, and capillary injury [15,234]. The development of ARDS is well documented in critically ill polytrauma patients, particularly in those with ISS > 25, traumatic brain injury, blood transfusion requirements, and significant orthopedic trauma such as long bone fractures [16,235]. Frequently, ARDS patients must be placed on prolonged mechanical ventilation with pressure support to prevent alveolar collapse, which itself can further contribute to issues of hyperoxia, nosocomial infection, and destruction of lung architecture in ventilator-induced lung injury. Gaining control of systemic inflammation is a key factor in improving patient outcomes in ARDS [236].

Neutrophil activity is a key aspect of ARDS, and their release of exosomes with pathogenic payloads has been demonstrated in other lung pathologies such as chronic obstructive pulmonary disease and bronchopulmonary dysplasia [15,237]. These pathogenic exosomes contain higher levels of surface-bound neutrophil elastase, which normally provides an antimicrobial role, but in aberrant expression, degrades lung architecture [237]. In patients with community acquired pneumonia that went on to develop ARDS, serum exosomes had increased expression of miR-146a, miR-27a, miR-126, and miR-155, and decreased miR-223 and miR-181b compared to patients that did not develop ARDS [204].

In a model of LPS-induced ALI, Jiang et al. showed that serum exosomes contained increased levels of miR-155, which stimulated NF-κB in macrophages, resulting in enhanced TNF-α and IL-6 production. When exosomes from ALI mice were infused into naïve mice, the disease state exosomes activated lung macrophages, expanded the M1 population, and induced pathological pulmonary inflammation [57]. Chen et al. identified 29 differentially expressed lncRNAs as part of the cargo from circulating monocyte-derived exosomes collected from ALI patients. The authors demonstrated that the co-culture of the human monoblastic cell line, U937, with the ALI exosomes augmented the secretion of IL-1β, IL-6, IL-15, IL-18, and TNF-α, suggesting M1 type macrophage activation. Moreover, they demonstrated that ALI monocyte-derived exosome preparations contained decreased levels of the lncRNA CLMAT3 which activates the CtBP2-p300-NF-κB transcriptional complex to induce robust levels of pro-inflammatory cytokines IL-1β, IL-6, and TNF-α. Overexpression of CLMAT3 reversed this effect, indicating the importance of the lncRNA in NF-kB modulation and inflammation in the setting of ALI [189].

Exosomes in the bronchoalveolar lavage fluid of ARDS patients contain proteins associated with apoptosis (caspases 9, 12), necroptosis (RIP3), and autophagy (microtubule-associated proteins 1A/1B light chain 3B) [205]. In the setting of hyperoxia-induced lung trauma, increased numbers of exosomes were released from lung epithelial cells in bronchoalveolar lavage fluid, containing increased levels of caspase-3. When the hyperoxia induced exosomes were administered to naïve mice, it caused pro-inflammatory effects, evidenced by increased numbers of alveolar macrophages and neutrophils in their bronchoalveolar lavage fluid. The lung epithelium-derived exosomes were also detected circulating in the serum, and peritoneal macrophages exposed to the serum exosomes expressed elevated levels of TNF-α and MIP-2 [206].

### 4.4. Sepsis

Sepsis, like SIRS, is diagnosed when the patient is experiencing a systemic inflammatory response but is differentiated by the presence of a known infection. The severity of the immune response to trauma, and concomitant CARS can place patients at risk for developing a severe infection. In an immunocompetent patient, infections are swiftly dealt with by the immune system with an appropriate inflammatory reaction ridding the body of the inciting pathogen. However, overstimulation of the immune system by both trauma and microbes creates the setting for an infection, leading to sepsis [238]. Increased ISS, decreased Glasgow Coma Scale (GCS) score, and pre-existing conditions place trauma patients at higher risk for developing sepsis [25]. If allowed to proliferate, local infections may progress to systemic infections attacking multiple organ systems. The systemic infection attacks the endothelial and epithelial barriers, leading to tissue damage and organ dysfunction, including ARDS and acute kidney injury [239]. A fatal complication of sepsis is disseminated intravascular coagulation (DIC), as the inflammatory cascade and inappropriate clot formation deplete coagulation factors, leading to spontaneous, and uninhibited bleeding [240]. Decreased systemic vascular resistance leads to low blood pressure and perfusion issues, and can cause septic shock [239]. These perfusion issues contribute to lactate being a recognized biomarker of sepsis since anaerobic glycolysis produces high levels of lactate. However, single biomarkers have proven less effective in predicting patient progression and outcome than combined panels of both pro- and anti-inflammatory targets [241].

The role of exosomes as biomarkers for assessing patient status and progression, and as therapeutic targets in septic patients has been previously reviewed [242,243]. In sepsis, exosomes carry critical mediators of the inflammatory response. Inhibition of exosome biogenesis and release with GW4869 (a pharmacological agent that blocks the ceramide-mediated inward budding of MVBs and mature exosome release) decreased the production of pro-inflammatory cytokines in a combined surgical trauma and endotoxin model, and improved survival [56]. Platelet-derived exosomes from septic patients carry active NADPH oxidase and NO synthase, contributing to the disruption of the endothelial barriers in the vasculature [213]. In an LPS model of sepsis, Alexander et al. were able to demonstrate functional transfer of exosomal miR-155 and miR-146a both in vitro and in vivo. The infusion of exosomes containing miR-155 in miR-155 -/- mice challenged with LPS resulted in increased levels of serum TNF-α and IL-6, whereas infusion of exosomes containing miR-146a in miR-146a -/- mice caused decreased serum levels of TNF-α and IL-6 [185]. Several other miRNAs are related to the pathogenesis of sepsis, particularly miR-27a, which induces an inflammatory response, is correlated with the developed of septic shock, and is differentially expressed in the plasma exosomes of sepsis patients [207]. Appiah et al. investigated intestinal epithelium-derived exosomes in the intestinal lavage fluid of a cecal ligation and puncture (CLP) sepsis model. Isolated exosomes from injured mice contained 360 differentially regulated miRNA and 190 unique miRNAs compared collections obtained from healthy controls. Injection of septic exosome preparations into the lumen of healthy ileum resulted in downregulation of TNF-α, IL-1β, IL-6, IL-17A, and IL-22, while treatment of inflamed ileum resulted in suppressed TNF-α and IL-17A activity, suggesting immunosuppressive activity [208]. Using a CLP model of sepsis, Wu et al. identified six miRNAs, miR-16, miR-17, miR-20a, miR-20b, miR-26a, and miR-26b, with increased expression in serum exosomes eight hours after injury [209]. Additionally, in a CLP model, Alkhateeb et al. found an 18-fold increase in the lncRNA HOXA transcript antisense RNA, myeloid-specific 1 (Hotairm1) in late sepsis (≥ day 5) Gr1^+^CD11b^+^MDSC-derived exosomes in a CLP model. In early sepsis (< day 5), the protein S100A9 was mostly confined to the cytosol, however, Hotairm1 causes a nuclear translocation of S100A9 in late sepsis, resulting in an immunosuppressive phenotype of Gr1^+^CD11b^+^MDSCs. Assessment of plasma exosomes in human sepsis patients also showed increased Hotairm1 [212]. Real et al. identified 28 differentially expressed exosomal miRNAs in sepsis patients both at the time of enrollment in the study and seven days later. Additionally, 35 differentially expressed plasma exosomal miRNAs were linked to patient outcome. Analysis of the pathways influenced by these miRNAs identified targets involved with inflammatory responses, particularly IL-6 signaling, acute phase response signaling, NF-κB signaling, and IL-10 signaling [210].

In a CLP model of sepsis, Gao et al. demonstrated that serum exosomes from septic mice obtained 2 h post injury contain significantly elevated levels of pro-inflammatory cytokines IL-1β, IL-2, IL-6, and TNF-α whereas the lymphocyte chemoattractants CCL2 (MCP-1) and CCL3 (MIP-1α) and anti-inflammatory cytokines IL-4 and IL-10 were not increased until 12 h and 24–48 h, respectively. The elevations of cytokines and chemokines in exosomes had different dynamics than their soluble counterparts in serum, suggesting a unique role for exosomal cargo in sepsis [214]. Reithmair et al. found a similar pattern in the miRNA cargo of exosomes, where intracellular, exosomal, and serum sepsis-associated miRNAs were compartment specific. While miR-27b was increased in blood cells, serum exosomes, and unfractionated serum, miR-125b elevations were unique to serum exosomes. The only other similarity existed in the upregulation of five miRNAs, miR-21-5p, miR-30a-5p, miR-100-5p, miR-122-5p, and miR-193a-5p, in both serum exosomes and unfractionated serum. Elevation of three exosomal miRNAs, miR-30a, miR-125b, and miR193a, correlated with disease severity and survival prediction [211].

### 4.5. Multi-Organ Dysfunction Syndrome and Multi-Organ Failure

Advances in trauma care have resulted in increased survival of patients after initial insults. However, these patients can experience delayed complications resulting from infection, hemorrhage and reperfusion injury, and dysregulated inflammation [23]. Multiple organ failure is a leading cause of delayed mortality in trauma patients [17,220]. The development of MOF occurs days to weeks after an inciting event (Figure 2) [112]. Major risk factors for the development of MOF in trauma patients includes ISS ≥ 16, age > 45 years, and male sex [244]. Injury patterns and interventions associated with a high ISS and increased risk of MOF are severe abdominal trauma, multiple fractures, severe thermal injuries, infection, and massive blood transfusions [21,112]. Transient multiple organ dysfunction may be present post-injury and during the resuscitation phase out to 48 h due to a hyperinflammatory response, but does not always progress to MOF [245]. The models for the development of MOF progression from SIRS are either “one hit” or “two hit”. In the “one hit model,” patients experience such severe trauma that SIRS rapidly progresses and leads to early MOF. In the “two hit” model, initial injury and under-resuscitation prime injured patients in a SIRS-like state to progress to MOF when exposed to a secondary insult such as surgery or other procedural resuscitation techniques [111,232]. This phenomenon can complicate surgical decision-making attempting to balance the resuscitative benefits of early appropriate care against the deleterious effects of surgery-induced inflammatory reactions. The development of SIRS, MODS, and MOF exist on a continuum, where recognition of these conditions and timely intervention can prevent progression to the next stage. The use of anti-cytokine and immunomodulation therapies in prevention of post-operative SIRS has not achieved success, and prevention of patient progression to MOF is regarded as the most effective treatment [17,109,232]. The clinical parameters for differentiating between the stages are similar, and progression may be subtle, necessitating identification of specific markers [21].

Research into the role of exosomes and their cargo specifically as they relate to the development of MODS and MOF post-injury are limited. Im et al. report an association between the level of plasma exosomes and Sequential Organ Failure Assessment (SOFA) in the prognosis of critically ill patients with sepsis, but did not profile the contents of the exosomes [246]. Murao et al. reviewed the impact of exosomes in end-organ damage in sepsis contributing to MODS, emphasizing the impact of exosomal delivery of sepsis-induced inflammatory mediators on the lungs, kidneys, liver, cardiovascular system, and central nervous system [247]. Examining the systemic inflammatory response to sepsis offers a potential framework for examining the impact of exosomal communication in traumatic injury, as PAMPs and DAMPs act on similar receptors. The study of MODS and MOF is largely a study of overwhelming and uncontrolled inflammation. As highlighted in the previous section, exosomal cargo has a significant role in inter-cellular communication of immune cells and the development and resolution of inflammation. Hemorrhage necessitates massive transfusions, and a major consequence of uncontrolled infection is sepsis, which has been discussed, but fracture and thermal injuries are also associated with unique exosomal cargo that may portend inflammatory progression and MODS or MOF development.

### 4.6. Fracture Healing

While exosomes regularly participate in bone homeostasis and remodeling, they also participate in resolution and potentiation of disease states in the skeletal system [36,39,248,249]. In a CD9 -/- mouse model, fracture healing was delayed, but rescued by injection of MSC-derived exosomes. Within these exosomes, miR-21, which is known to promote osteogenic differentiation of MSCs, was highly expressed [249]. Consistent with some degree of inflammation necessary in the setting of injury, miR-21 is also an NF-κB-dependent miRNA that is upregulated in response to peripheral blood mononuclear cell stimulation with LPS [250]. Currently, research on exosomes and bones outside of the setting of osteoporosis is limited, but since exosomal cargo is known to have a role in the setting of fracture, additional studies in the setting of fracture in patients with healthy bone density may yield more benefits to the study of severe trauma.

### 4.7. Thermal Injury

Large surface area burns drive a systemic inflammatory response from massive tissue injury accompanied by major fluid loss through local edema and barrier disruption, liberated tissue breakdown products, and hypovolemia-induced cardiac dysregulation. While catecholamines and corticosteroids are the primary mediators of metabolic dysfunction in burn patients, inflammatory mediators such as IL-1, IL-6, and TNF have also been implicated, particularly in contributing to the severity of dysfunction and catabolism [251,252]. Muscle catabolism impairs the immune response and wound healing, and places patients at risk for complications and death [252]. Qin et al. found that there was differential expression of 31 proteins in the serum exosomes of burn patients. Of particular note in their findings was the upregulation of ITGA2B and ITGB3 in the burn group, as these proteins act in the PI3K/AKT signaling pathway, which promotes cell proliferation and growth [253]. Wound healing, especially cutaneous, is critical in a burn patient, to restore the body’s barrier to fluid loss and infection. Persistence of inflammation can delay wound healing, but anti-inflammatory miRNAs such as miR-124a and miR-125b found in exosomes have the ability to manipulate expression of inflammatory cytokines in the wound microenvironment to create a more favorable setting for repair [254]. The role of exosomal cargo as a therapeutic option, especially MSC-derived exosomes, in the setting of burn has been more extensively studied than the profiles of exosomes in injured burn patients. Li et al. determined in a rat burn model that human umbilical cord MSC-derived (hucMSC) exosomal delivery of miR-181c was able to attenuate the immune response to burn by decreasing TLR4 expression and NF-κB/p65 activation [255]. In a rat burn model, Zhang et al. demonstrated that hucMSC-derived exosomes carrying Wnt4 enhanced cutaneous wound healing [256].

## 5. Conclusions

Previous research has demonstrated the role of exosomes and their cargo in the development, control, and resolution of inflammation. Characterization of their role in inflammatory immunological processes indicates they are pivotal in many pathological states. Information is lacking in how these extracellular messengers contribute in the context of severe trauma, and the subsequent uncontrolled inflammation that follows. Further investigation into the exosome profiles following polytraumatic injuries may provide valuable insight into the early stages of severe systemic inflammation and provide opportunities for early intervention. Identification of inciting biomolecular events for conditions like SIRS is critical for preventing delayed patient deterioration to ARDS, sepsis, or MOF after initial resuscitation. While systemic markers of inflammation, such as IL-6 are already widely used, the unique cargo of exosomes may help to differentiate the effects of trauma upon the body, rather than using broad markers of systemic dysfunction. Secondly, indication that markers of inflammation are detected earlier within circulating exosomes compared to their free, non-exosomal counterparts in circulation, provides potential opportunity of earlier intervention than in current practices. Exosomal cargo appears to reflect local tissue and systemic pathology, thereby affecting remote organ function, and potentially functioning as a critical read-out of post-traumatic damage. Put together, these circulating, membrane-bound vesicles offer a new method of characterizing trauma-related “fingerprint molecular signatures,” with the opportunity to enhance the efficacy of clinical decision-making tools and precision medicine. The observed association between inflammation and change in nature or expression levels of specific exosomal cargo is the fundamental step for identifying possible novel biomarkers of inflammatory-based pathologies, diseases, and clinical sequelae.

## Figures and Tables

**Table 1:** Summary table of bioactive cargo of exosomes in trauma-associated inflammatory complications. This table specifically highlights micro-RNA (miRNA), messenger RNA (mRNA), and proteins found in exosomes associated with ischemic reperfusion injury (IRI), acute respiratory distress syndrome (ARDS), and sepsis.

Trauma Associated Condition	Bioactive Material	Exosomal Cargo	Literature
IRI	miRNA	miR-23a	Crouser et al., 2021 [197]
		miR-24	Minghua et al., 2018 [198]
		miR-1, miR-133a, miR-24, miR-210, miR-133b	Emanueli et al., 2016 [199]
		miR-16-5p, miR-24-3p, miR-200c-3p, miR-9a-5p, miR-141-3p, miR-200a-3p, miR-429	Sonoda et al., 2019 [200]
		miR-1, miR-208, miR-499, miR-133	Chen et al., 2019 [201]
	mRNA	Upregulated: Vsig1, Top1, Ccbp2, 0610010K06Rik, Krit1, D230019N24Rik, Amy2a1, Lba1, Zfp385c, 2700057C20Rik, Ptar1, Smad3, 2810002D19Rik, Phf6, Hsd17b11, 6720457D02Rik, Yipf7, Mep1a, Sox15, 4930473M17Rik	Eldh et al., 2010 [202]
		Downregulated: Ctnna1, Pigq, Cct2, Rfc4, Gnas, Ttc3, Laptm5, Gabarapl1, Ipo4, Dnpep, Lmna, Ssr3, Qars, Gsn, Arap3, Med22, Csnk1d, Coro7, Lasp1, Ric8	
	Protein	C3 propeptide, PK-120 precursor, alpha amylase one precursor, beta-enolase isoform 1, adenylosuccinate synthetase isozyme 1	Yang et al., 2015 [203]
		Hsp70	Zheng et al., 2018 [193]
ARDS	miRNA	Upregulated: miR-146a, miR-27a, miR-126, miR-155	Wu et al., 2019 [204]
		Downregulated: miR-223, miR-181b	
		miR-155	Jiang et al., 2019 [57]
	lncRNA	Upregulated: AOC4P, BCAR4	Chen et al., 2020 [189]
		Downregulated: CLMAT3, MIAT	
	Protein	caspase 12, caspase 9, RIP3, microtubule associated proteins 1A/1B light chain B3	Kim et al., 2019 [205]
		caspase 3	Moon et al., 2015 [206]
Sepsis	miRNA	miR-155, miR-146a	Alexander et al., 2015 [185]
		miR-27a	Hashemian et al., 2020 [207]
		miR-19a, miR-21a, miR-22, miR-27a, miR-103-2, miR-107, miR-126a, miR-146b, miR-182, miR-200b, miR-203, miR-762	Appiah et al., 2020 [208]
		miR-16, miR-17, miR-20a, miR-20b, miR-26a, miR-26b	Wu et al., 2013 [209]
		Upregulated: let-7b-5p, let-7c-5p, miR-122-5p, miR-1227-3p, miR-125b-5p, miR-1260a, miR-1262, miR-1267, miR-1290, miR-1298-5p, miR-1300, miR-140-3p, miR-16-5p, miR-1825, miR-192-5p, miR-193a-5p, miR-194-5p, miR-195-5p, miR-19a-3p, miR-25-3p, miR-30a-5p, miR-320a, miR-320b, miR-363-3p, miR-486-5p, miR-518d-3p, miR-519b-3p, miR-520d-3p, miR-532-3p, miR-548a-3p miR-548c-3p, miR-597-5p, miR-618, miR-625-3p, miR-636, miR-645, miR-720, miR-758-3p, miR-770-5p, miR-885-5p, miR-886-5p, miR-92a-3p, miR-99b-3p	Real et al., 2018 [210]
		Downregulated: miR-127-3p, miR-146a-5p, miR-151a-3p, miR-186-5p, miR-18a-5p, miR-199a-3p, miR-221-3p, miR-26a-5p, miR-28-5p, miR-301a-3p, miR-328, miR-331-3p, miR-335-5p, miR-339-3p, miR-340-5p, miR-340-3p, miR-590-3p, miR-628-5p, miR-744-5p	
		miR-16, miR-17, miR-20a, miR-20b, miR-26a, miR-26b	Wu et al., 2013 [209]
		miR-27b, miR-125b, miR-21-5p, miR-30a-5p, miR-100-5p, miR-122-5p, miR-193a-5p	Reithmair et al., 2017 [211]
	lncRNA	Hotairm1	Alkhateeb et al., 2020 [212]
	Protein	NADPH, NO synthase	Gambim et al., 2007 [213]
		IL-1β, IL-2, IL-6, TNF-α, IL-4, IL-10, CCL2, CCL3	Gao et al., 2019 [214]

## Disclaimer

The contents of this publication are the sole responsibility of the author(s) and do not necessarily reflect the views, opinions, or policies of Uniformed Services University (USU), The Henry M. Jackson Foundation for the Advancement of Military Medicine, Inc., the Department of Defense (DoD), the Departments of the Army, Navy, or Air Force. Mention of trade names, commercial products, or organizations does not imply endorsement by the U.S. Government. This work was prepared by a military (S.A.W., B.W.H.) or civilian (T.A.D.) employee of the US Government as part of the individual’s official duties and therefore is in the public domain and does not possess copyright protection.
